# Caloric restriction induces heat shock response and inhibits B16F10 cell tumorigenesis both *in vitro* and *in vivo*

**DOI:** 10.18632/aging.100732

**Published:** 2015-04-05

**Authors:** Marta G. Novelle, Ashley Davis, Nathan L. Price, Ahmed Ali, Stefanie Fürer-Galvan, Yongqing Zhang, Kevin Becker, Michel Bernier, Rafael de Cabo

**Affiliations:** ^1^ Translational Gerontology Branch, National Institute on Aging, National Institutes of Health, Baltimore, MD 21224, USA; ^2^ Gene Expression and Genomics Unit, National Institute on Aging, National Institutes of Health, Baltimore, MD 21224, USA; ^3^ Department of Physiology, CIMUS, University of Santiago de Compostela-Instituto de Investigación Sanitaria, 15782 Santiago de Compostela, Spain; CIBER Fisiopatología de la Obesidad y Nutrición (CIBERobn), 15706 Santiago de Compostela, Spain

**Keywords:** caloric restriction, heat shock, stress response, tumorigenesis, aging

## Abstract

Caloric restriction (CR) without malnutrition is one of the most consistent strategies for increasing mean and maximal lifespan and delaying the onset of age-associated diseases. Stress resistance is a common trait of many long-lived mutants and life-extending interventions, including CR. Indeed, better protection against heat shock and other genotoxic insults have helped explain the pro-survival properties of CR. In this study, both *in vitro* and *in vivo* responses to heat shock were investigated using two different models of CR. Murine B16F10 melanoma cells treated with serum from CR-fed rats showed lower proliferation, increased tolerance to heat shock and enhanced HSP-70 expression, compared to serum from *ad libitum*-fed animals. Similar effects were observed in B16F10 cells implanted subcutaneously in male C57BL/6 mice subjected to CR. Microarray analysis identified a number of genes and pathways whose expression profile were similar in both models. These results suggest that the use of an *in vitro* model could be a good alternative to study the mechanisms by which CR exerts its anti-tumorigenic effects.

## INTRODUCTION

Aging is a complex multifactorial process, whereby organisms undergo major cell degeneration and loss of function. During aging, irreversible and deleterious processes are triggered by accumulation of damaged cellular macromolecules [1, 2]. Several theories have been proposed to explain these processes [3, 4], but the exact molecular mechanisms behind aging remains unknown. Cellular damage may result from oxidative stress, toxic metabolic byproducts, endoplasmic reticulum stress and mitochondrial unfolded protein responses, or exposure to heat stress, among others. Several heat shock proteins (HSP) function as molecular chaperones by preventing misfolding and aggregation of other proteins. This induction of cytoprotective responses promotes longevity [5, 6]; conversely, aging is associated with down-regulation in HSP expression in neuronal tissue, skeletal and cardiac muscle, and the liver [7, 8]. Stimulation of HSP synthesis has been suggested as a viable strategy to counteract the negative effects of aging and eliciting a ‘low-grade’ stress response may help organisms live longer and improve their survival [9].

More than 8 decades ago, McCay and colleagues observed that severe reduction in calorie intake while maintaining sufficient micronutrient levels for optimum health resulted in lifespan extension [10]. Since then, numerous studies have reported that lifelong caloric restriction (CR) extends mean and maximum lifespan and delays age-associated diseases in a wide variety of species [11, 12]. Many of the beneficial effects of CR are mediated by altering the expression of several HSPs, notably Hsp70, and the activation of heat shock transcription factor 1 [13-15]. In this context, our group has demonstrated that exposure of HepG2 cells to human serum from CR participants conferred significant cytoprotection against heat stress [7]. Moreover, cells treated with human serum from CR volunteers trigger a transcriptional up-regulation of numerous genes and pathways implicated in stress resistance through activation of the transcription factor NF-E2-related factor (NRF2) [16]. NFR2 plays a key role in maintaining homeostasis during oxidative stress and exposure to carcinogens by coordinately regulating the expression of antioxidants and detoxification enzymes [17] that boost protection against cancer [18].

The anti-tumorigenic properties of CR on spontaneously arising tumors and in experimental cancer models are well-documented [19]. For example, 15 days of 40% CR significantly reduces the growth of brain tumors in mice by reducing angiogenesis and increasing tumor cell apoptosis [20]. The combination of fasting and chemotherapy retards the growth of human breast cancer tumors in mice [21] and delays the progression of pancreatic cancer lesions in a mouse model [22]. The use of the mouse as an experimental tool in cancer research is cumbersome, time-consuming and expensive, and, therefore, has compelled us to explore an alternative approach to study anti-cancer therapies.

In this manuscript we present a new approach to investigate a central mechanism by which CR activates a stress response pathway to combat tumorigenesis. The stress response of murine B16F10 melanoma cells maintained in culture medium supplemented with serum from rats fed CR and *ad libitum* (AL) diet was evaluated and compared to that of mice injected with B16F10 melanoma cells and maintained on either CR or AL. In the latter experimental model, mice were subjected to heat stress followed by the monitoring of a melanoma-specific Hsp70 reporter expression. These results combined with microarray analysis illustrated alteration of a common set of cancer-related genes using in *vitro* and *in vivo* testing.

## RESULTS

### Growth rate and heat shock response of B16F10 melanoma cells maintained in serum from CR-fed animals

**Figure 1:**
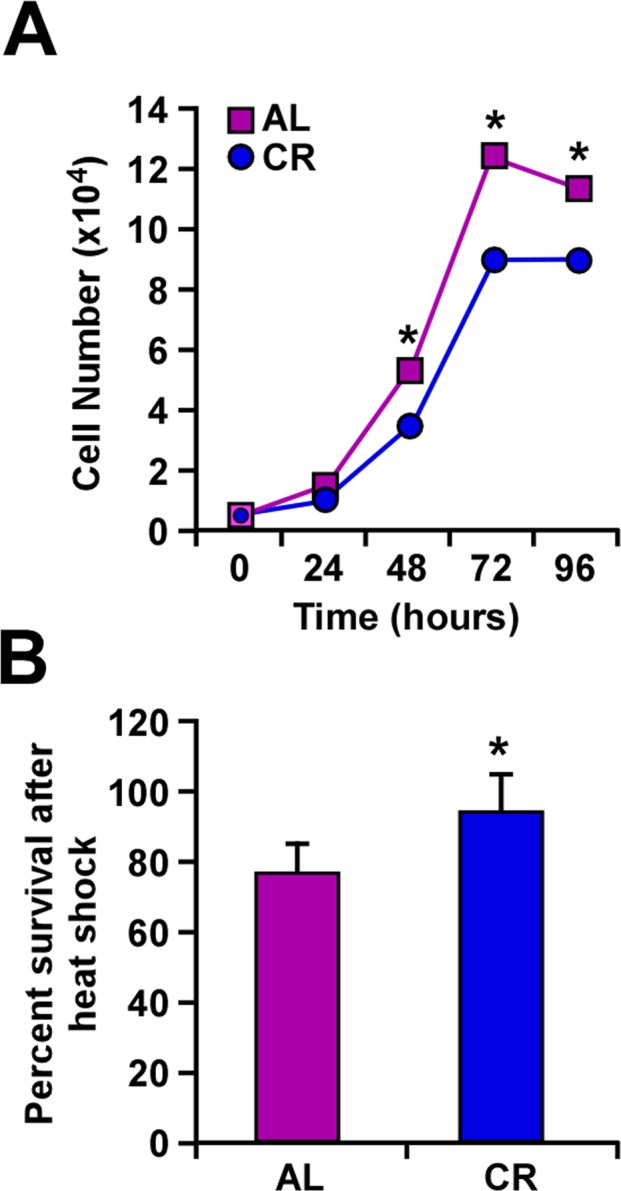
Caloric restriction slows cellular growth and improves response to heat shock (**A**) B16F10 melanoma cells were maintained in culture with serum from AL- and CR-fed rats over a period of 96 h. The number of cells was counted at 24-h intervals. (**B**) Percent of cells surviving a 1-h treatment at 45°C when maintained in culture with serum from either AL- or CR-fed rats. Data are represented as the mean ± SEM. *, p< 0.05.

Proliferation of B16F10 melanoma cells was carried out in the presence of serum from rats fed either AL or CR diet. A significant reduction in cell growth was observed following incubation with CR serum as compared to AL serum controls (Fig. 1a). Exposure of B16F10 cells to heat stress (45°C) for 1 h caused a significant difference in survival depending on whether these cells were maintained in CR or AL serum (Fig. 1b). Our observations that CR serum decreased heat-dependent cellular cytotoxicity support previously published results from this laboratory [23].

### Reduction in the number and size of tumors in mice on caloric restriction

**Figure 2:**
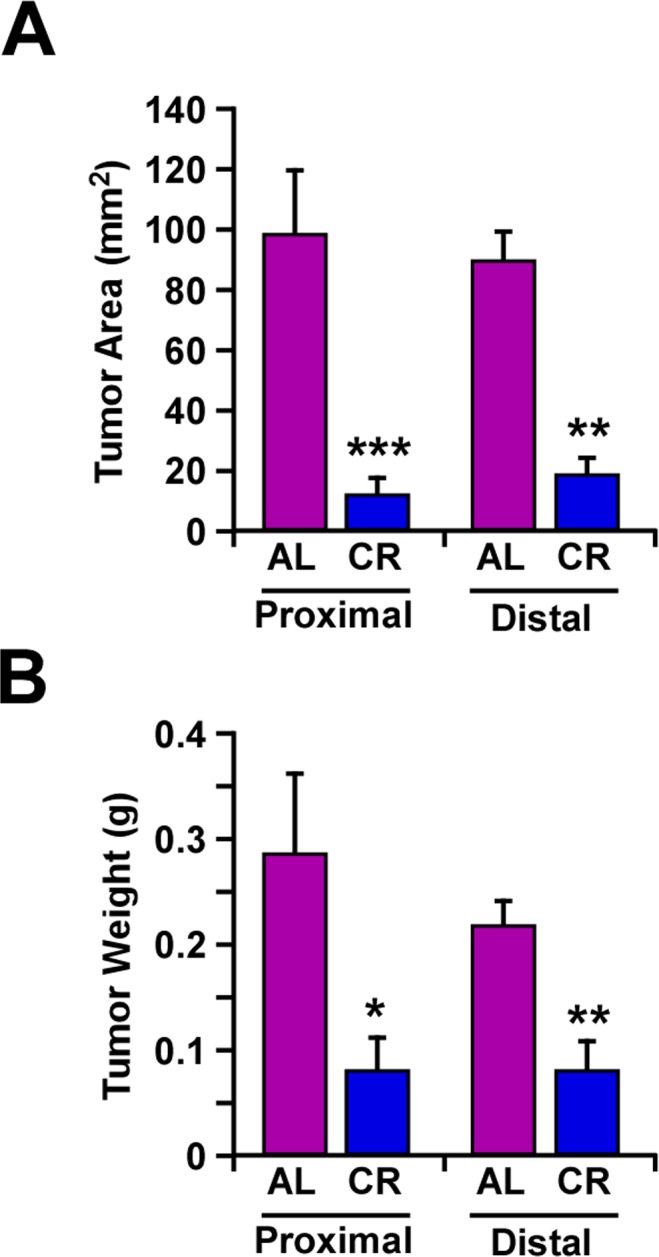
Caloric restriction decreased melanoma tumor growth *in vivo* In AL- and CR-fed mice, mouse tumor xenografts were formed by implanting B16F10 melanoma cells, at the periscapular region (proximal area) and in the lower back over the hip (distal area). Tumor area (mm^2^) (**A**) and individual tumor weight (g) (**B**) were determined after 14 days. Data are represented as the mean ± SEM. n=10/per group. *, p< 0.05, **, p<0.01, *** p<0.001.

To evaluate the effect of CR on tumor growth *in vivo*, mouse B16F10 melanoma cells that were stably transfected with Hsp70-GFP plasmid, were implanted subcutaneously in male C57BL/6 mice fed either a CR or AL diet. A significant decrease in the size and weight of tumors was observed in CR-fed mice along with delayed tumor growth both in the periscapular region and lower back area (Fig. 2).

### Heat stress-mediated induction of Hsp70 expression both in *in vitro* and *in vivo* models

**Figure 3:**
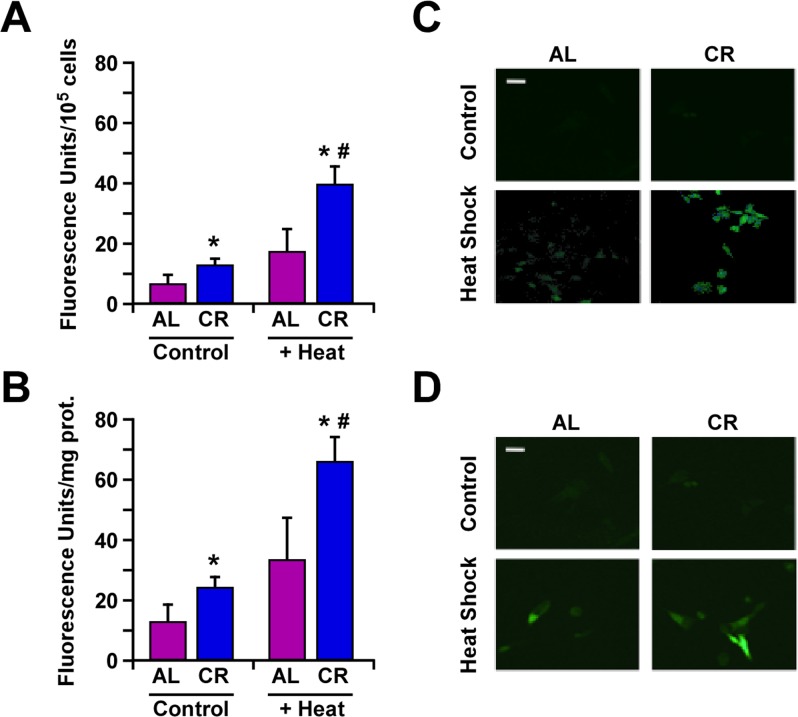
Caloric restriction improves protection against heat shock through increased expression of Hsp70 (**A**) B16F10 melanoma cells stably transfected with a plasmid encoding GFP-tagged Hsp70 construct were maintained in medium supplemented with 10% serum from AL- or CR-fed rats and then subjected or not to heat shock stress for 45 min. Bars represent fluorescence intensity per 10^5^ cells. Cell culture experiments were performed as three or more replicates. (**B**) B16F10 tumor xenografts from mice fed either AL or CR diet were subjected to heat shock stress for 45 min and sacrificed after 4h. Bars represent fluorescence intensity per mg of tumor proteins; n=10 per group. Data, obtained by fluorimetery, are represented as the means ± SEM. *, p <0.05 *vs*. AL group; #, p<0.05 *vs*. CR group. (**C**) Images of B16F10 cells and (**D**) tumor cells depicting GFP fluorescence were detected by confocal microscopy. White bar, 20 μm.

Changes in HSP expression play an important role in the ability of cells to respond to environmental stressors. Earlier work has shown an elevation in Hsp70 expression in B16F10 melanoma cells cultured with serum from CR-fed animals [23]. Here, we compared the effects of CR alone, heat shock alone or the combination ‘CR + heat shock’ using B16F10 melanoma cells stably expressing GFP-tagged Hsp70 construct under the control of rat *hsp70.1* promoter [24]. The results indicate that the heat-mediated induction of Hsp70 expression was significantly higher when B16F10 cells and tumor-bearing animals were subjected to CR (Fig. 3).

### Microarray analysis of B16F10 melanoma cells used in *in vitro* and *in vivo* settings

**Figure 4:**
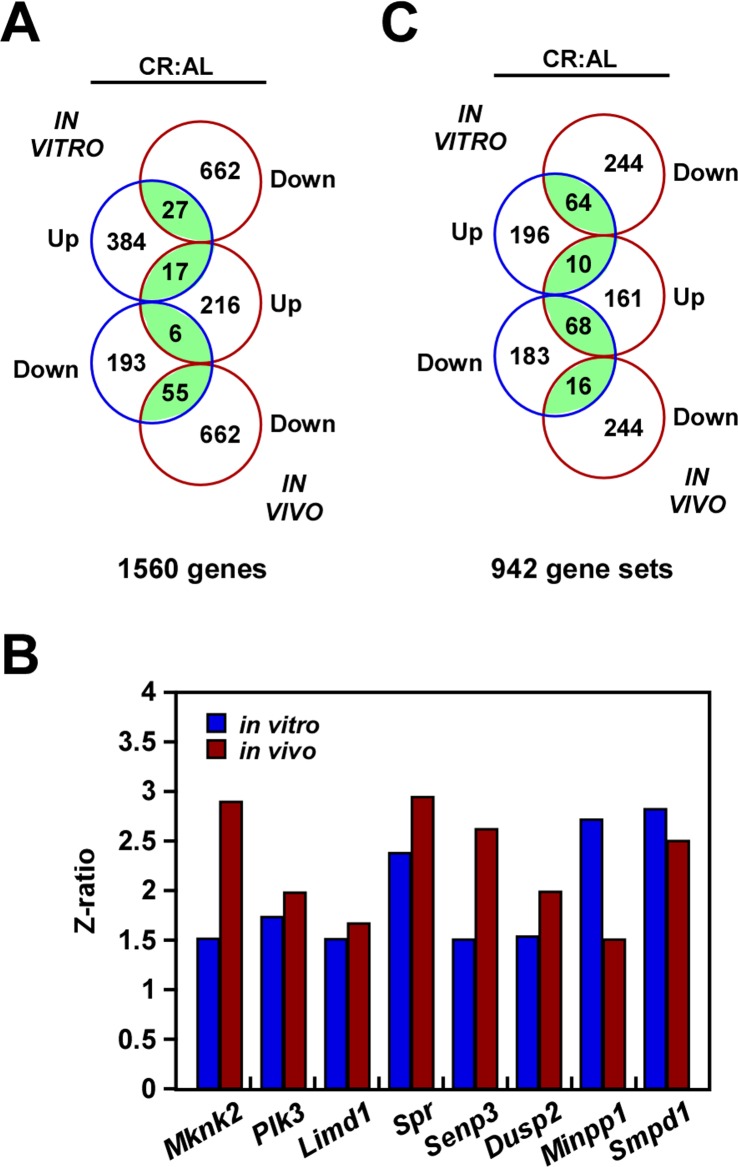
Gene expression profiling in response to caloric restriction (**A**) Venn diagram showing the overlap of gene **transcripts** with significant change in expression in the CR *versus* AL pairwise comparisons by both B16F10 melanoma cells growing in culture (*in vitro*) and as tumor xenografts (*in vivo*). (**B**) Effect of CR on the expression of a select group of transcripts. (**C**) Venn diagram showing the overlap of gene sets with significant change in expression in the CR *versus* AL pairwise comparisons.

DNA microarray analysis was performed to compare the global transcriptional effect of CR in B16F10 melanoma cells either grown in culture or implanted in mice. Principal Component Analysis (PCA) revealed inherent *in vivo* and *in vitro* differences that must be taken into account when comparing the impact of CR in gene expression profile. Nevertheless, Venn diagram indicated that both models shared 55 up-regulated and 17 down-regulated transcripts, which were significantly enriched in the CR versus AL pairwise comparisons (Fig. 4a, Supplemental Table 1). Among these shared transcripts, MAP Kinase Interacting Serine/Threonine Kinase 2 (*Mknk2*) [25], polo-like kinase 3 (*Plk3*) [26] and LIM Domains Containing 1 (*Limd1*) [27] are implicated in tumorigenesis, whereas *Spr* and *Semp3*, which encode for Sepiapterin Reductase and SUMO1/Sentrin/SMT3 Specific Peptidase 3, are involved in stress response [28, 29]. DUSP2 is an important member of the dual-specificity protein phosphatase subfamily, which is implicated in inflammatory response and reported to be upregulated both with CR and heat shock [30]. Moreover, there is an increased expression of *Minpp1*, which encodes for Multiple Inositol Polyphosphate Phosphatase 1. This phosphatase is induced in response to heat shock, osmotic and oxidative stress conditions, thereby contributing to the regulation of ER stress and apoptosis [31]. Finally, upregulation of *Smpd1* (Sphingomyelin phosphodiesterase 1, also known as ASM) was also observed in both experimental models with CR (Fig. 4b). Its activity is expressed at high levels in cancer cells under the control of an inducible expression of hsp70.1 protein [32]. Using parametric analysis of gene set enrichment, 26 gene sets were identified whose expression levels were significantly altered in the same direction by CR in both experimental models (Fig. 4c, Supplemental Table S2).

## DISCUSSION

The aging process involves multiple physiological mechanisms and represents one of the main risk factors for several human pathologies, such as cancer, diabetes, and cardiovascular disease. Dietary CR retards the aging process and age-related disease pathogenesis [33, 34], and many studies have tried to elucidate the exact mechanism(s) by which CR acts (reviewed in [35]). Our work demonstrates that CR significantly decreases tumor cell proliferation, in agreement with previous studies [36, 37], and this phenomenon takes place whether B16F10 melanoma cells were cultured with serum from CR-fed animals or these tumor cells were implanted in CR-fed mice. Moreover, the process of tumorigenesis was significantly decreased in CR-fed animals after heat stress. Hsp70 is one of several heat-shock proteins implicated in the regulation of cancer cell growth. HSPs sustain tumor survival and drive tumor growth [38]; however, induction of Hsp70 family members results in cellular protection against un-favorable environmental conditions, including elevated temperatures, oxidative stress, exposure to heavy metals, proteasome inhibitors, and infection [39]. CR has been previously shown to restore the ability of cells to mount a heat shock response through increase in Hsp70-mediated thermotolerance [40], an observation that was confirmed in the present study. Moreover, B16F10 melanoma cells subjected to heat shock stress showed greater survival when maintained in CR serum as compared to serum from AL-fed animals. It would appear that heat stress and CR acted cooperatively to enhance cell survival, possibly via activation of the deacetylase SIRT1 [40, 41]. It is interesting to note that the combination of heat stress with CR caused a synergistic increase in Hsp70-GFP expression when compared to either condition alone both *in vitro* and *in vivo*.

Microarray results reinforce the idea that despite significant genome-wide gene expression variation between the two experimental models, the expression profile of several transcripts implicated in tumorigenesis and stress response exhibited a comparable pattern, whether B16F10 melanoma cells were cultured in CR serum or implanted in mice fed a CR diet. Although this *in vitro* model is quite distant from a physiological setting, it displayed a number of molecular pathways similar to the ones observed *in vivo*.

In conclusion, our findings indicate that the impact of CR on the regulation of several pathways implicated in tumorigenesis on an *in vivo* model of heat stress response can be replicated *in vitro* using tumor cells incubated with serum from CR-fed animals. The idea that hormones and nutrients present in serum, whose levels are altered during CR, are involved in homeostasis control mechanisms, including the aging process, has been suggested [7, 16, 23]. These results support the notion that *in vitro* testing may be well suited for the study of molecular aspects of CR that have not been elucidated yet.

## MATERIALS AND METHODS

### Animals and Dietary Manipulation

The mice were single-housed in duplex caging in a room maintained at a constant temperature (20-22 °C) and humidity (30-70%) in a light:dark 12:12-h schedule, according to established animal protocols and NIH guidelines. Male C57BL/6 mice (3 month old) were fed on a standard purified mouse diet (NIH-31) *ad libitum* (AL; n=10) or maintained on a 40% calorie restriction regimen (CR; n=10) during six weeks. Body weight and food intake was recorded weekly (supplemental figure 1 a, b).

### Cell culture

B16F10 melanoma cells (ATCC^®^ CRL-6475^™^) were purchased from American Type Culture Collection (Manassas, VA); they were cultured in Dulbecco's Modified Essential Medium (DMEM) supplemented with 10% fetal bovine serum and penicillin/streptomycin (Gibco, Gaithersburg, MD) under standard cell culture conditions. Cells were incubated in media with 10% serum from AL- or CR-fed rats (as described previously [23]). Briefly, serum was obtained from overnight fasted, anesthetized 6-month-old male Fisher 344 rats from three different cohorts. The blood collection took place between 7-11:00 a.m. After a 1-h incubation in a water bath at 45°C, cells were trypsinized, washed twice with phosphate-buffered saline (Invitrogen, Grand Island, NY), and then seeded at 1.5×10^5^ cells/well in 96-well plates. Cell proliferation assays were carried out during 96 h by the addition of a tetrazolium salt solution, WST-8, to each well according to the manufacturer's protocol (Dojindo, Indianapolis, IN). The absorbance of the formazan dye formed was measured at 450nm using the Perkin Elmer HTS 7000 Plus BioAssay reader.

### Heat shock treatment of B16F10 melanoma xenografts in vivo

One month into the study, mice were injected with 1×10^6^ B16F10 melanoma cells stably transfected with a plasmid containing *GFP* gene linked to rat stress-inducible *hsp70.1* gene promoter [24] in the periscapular region (proximal) and in the lower back over the hip (distal area). After a two-week period, five mice were randomly chosen from each group and placed in a tumor hyperthermia induction chamber (THIC) constructed in our facility. Mice were anesthetized with isoflurane droplets in a closed chamber prior to being placed in the THIC and maintained under anesthesia for the reminder of the experiment. Two membranous tubes filled with pre-warmed water were placed over the proximal (45°C) and distal (27°C) tumors of the anesthetized mice *(supplemental figure 1 c, d)*. The water temperature was maintained throughout the duration of the experiment. After a 45-min heat treatment, the mice recovered for 4 h and then euthanized by cervical dislocation, according to the AAALAC guidelines.

### Melanoma tumor growth *in vivo*

Tumor xenografts were formed by implanting murine B16F10 melanoma cells at the periscapular region and in the lower back of AL- and CR-fed mice. Fourteen days later, animals were euthanized and tumors were excised for the determination of the tumor area using a caliper. Tumor weight was also recorded.

### GFP fluorescence detection

Experiments were carried out as indicated, using B16F10 melanoma cells stably expressing a plasmid encoding GFP-tagged Hsp70 that were either maintained in culture or used as tumor xenografts in mice. GFP fluorescence was monitored using both a confocal microscope (Axiovert-200, Zeiss LSM 510) to obtain images and a fluorimeter (Perkin-Elmer LS-55 and HTS 7000 Plus BioAssay reader) to accurately quantify GFP expression levels, which were normalized per 10^5^ cells (in culture) or mg of tumor proteins.

### Microarray analysis

RNA was isolated from B16F10 melanoma cells maintained in culture and as tumor xenografts. For microarray analysis, RNA was processed, reverse transcribed, labeled and hybridized to Mouse 15K cDNA arrays and read on an Illumina BeadArray 500GX reader. Raw data were subjected to Z normalization to ensure compatibility using the formula: z(raw data)=[ln (raw data) – avg(ln(raw data))]/[std dev(ln (raw data))], where ln is natural logarithm, avg is the average over all genes of an array, and std dev is the standard deviation over all genes of an array (Cheadle et al., 2003). The Z ratio (between treatment A and B) is given by z(A)-z(B)/std dev. Individual genes with Z ratio > 1.5 in both directions, *P* value < 0.05, and false discovery rate > 0.3 were considered significantly changed. All raw data were deposited in the NCBI Gene Expression Omnibus under accession number GSE67430.

### Statistical analysis

Statistical analyses were performed using Microsoft Excel software (Microsoft Corp., Redmond, WA). Unpaired t-tests were used for all analyses. Statistical significance was established at *p*<0.05. Data are expressed as means ± standard error of the mean (SEM).

## SUPPLEMENTAL MATERIAL FIGURE AND TABLES






