# Supplementary material for: Caloric restriction induces heat shock response and inhibits B16F10 cell tumorigenesis both in vitro and in vivo
Source: Aging (Albany NY). 2015 Apr 5;7(4):233–9. doi: 10.18632/aging.100732 (PMC4429088; doi:10.18632/aging.100732)
Supplement: Supplementary file 1 [file aging-07-233-s001.pdf]

SUPPLEMENTAL MATERIAL

Please browse the full text version of this manuscript to see the Supplemental Tables 1 and 2.

Supplemental Table 1. List of genes with significant change in expression by both experimental models in response to CR compared to AL.

Supplemental Table 2. List of gene sets significantly enriched in both experimental models in response to CR.

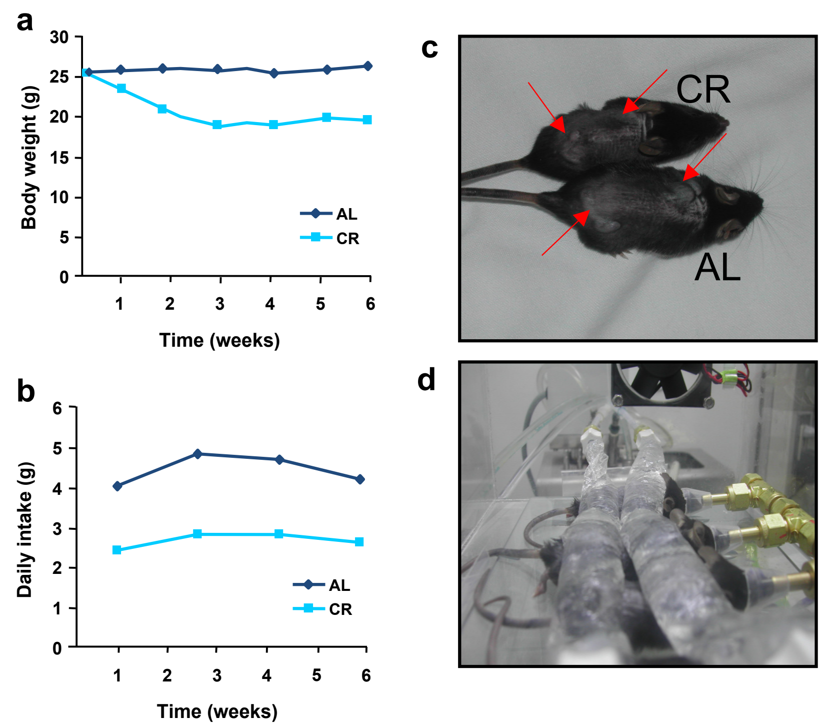

Supplemental Figure 1. Characterization of the animal model. (**a**) Body weight and (**b**) food consumption of mice fed an *ad libitum* (AL) or caloric restriction (CR) diet over the duration of the experiment. Data are represented as the mean ± SEM. (**c**) Image of C57BL/6 mice after injection of B16F10 melanoma cells. Red arrows denote location of the tumors on a CR- and AL-fed mouse. (**d**) Side view of mice placed in the tumor hyperthermia induction chamber.
