# Supplementary material for: Caloric restriction induces heat shock response and inhibits B16F10 cell tumorigenesis both in vitro and in vivo
Source: Aging (Albany NY). 2015 Apr 5;7(4):233–9. doi: 10.18632/aging.100732 (PMC4429088; doi:10.18632/aging.100732)
Supplement: Supplementary file 2 [file aging-07-233-s002.docx]

**Supplemental Table 1.**

**List of genes with significant change in expression by both experimental models in response to CR compared to AL.**

| **Gene Symbol** | **In vitro model (Z-ratio)** | **In vivo model (Z-ratio)** |
| --- | --- | --- |
| 1700019D03Rik  Mgll  Fkbp11  Srprb  0610008A10Rik  5730438N18Rik  Hras1  Sdf2l1  Spr  Mknk2  Vkorc1  Cdc42ep5  Crtap  Grn  Atp6v0a1  0610039N19Rik  Senp3  2700060E02Rik  BC008103  3110082I17Rik  Mgat4b  Fmip  Smpd1  C2  Bace2  Atp6v1c1  Eps8  D5Ertd33e  Snrpa  Syngr2  Epb4.1l4a  Jtb  Mmrp19  C78915  Psmb5  Mgat4b  Dusp2  Plk3  Stra13  Rac1  Supt3h  D19Ertd144e  Cln8  Tnfrsf12a  Mthfd1  E130307C13  Thoc4  Bcl7c  Limd1  Rwdd1  9430029K10Rik  Fus  Prss25  Aup1  Minpp1 | 2.70  3.46  1.62  1.84  2.81  2.81  2.95  2.98  2.38  1.53  2.04  3.25  1.69  1.58  1.92  1.67  1.51  2.47  2.16  1.84  3.24  1.67  2.83  3.03  4.88  2.43  2.13  3.77  2.87  3.78  1.78  1.89  2.37  2.16  3.07  3.38  1.55  1.74  1.88  2.44  2.28  1.97  2.18  2.97  2.19  2.13  2.19  1.56  1.51  3.57  2.64  2.27  2.11  3.93  2.72 | 4.56  4.07  3.86  3.66  3.58  3.56  3.11  3.02  2.95  2.90  2.84  2.80  2.71  2.65  2.64  2.63  2.63  2.62  2.56  2.56  2.55  2.55  2.51  2.45  2.42  2.40  2.39  2.34  2.26  2.25  2.20  2.20  2.13  2.12  2.06  2.06  2.00  2.00  1.97  1.97  1.96  1.93  1.83  1.83  1.80  1.77  1.74  1.69  1.68  1.67  1.65  1.64  1.62  1.59  1.52 |
| Foxj2  Cbx7  Nfkbiz  Hnrpdl  Mfap3  Cops7b  Stard4  Gas5  Acadsb  Arfip1  Clk1  4833420G17Rik  Sdc4  Slc40a1  Dbp  Tde2  Clcn3 | -1.60  -2.15  -2.62  -1.82  -2.52  -2.09  -1.69  -1.98  -2.21  -2.02  -2.30  -2.44  -2.49  -2.93  -2.14  -1.58  -3.06 | -1.53  -1.56  -1.64  -1.66  -1.68  -1.88  -2.35  -2.39  -2.46  -2.52  -2.58  -2.69  -2.77  -3.47  -3.72  -3.84  -4.32 |

All transcripts were statistically significant with Z-ratio >1.5 in both directions, p < 0.05 and false discovery rate < 0.3 (see Experimental Procedures for additional details).
