# Supplementary material for: Caloric restriction induces heat shock response and inhibits B16F10 cell tumorigenesis both in vitro and in vivo
Source: Aging (Albany NY). 2015 Apr 5;7(4):233–9. doi: 10.18632/aging.100732 (PMC4429088; doi:10.18632/aging.100732)
Supplement: Supplementary file 3 [file aging-07-233-s003.docx]

**Supplemental Table 2.** **List of gene sets significantly enriched in both experimental models in response to CR.**

| Pathway | n genes | Z-score | P value | fdr | Z-score | P value | fdr |
| --- | --- | --- | --- | --- | --- | --- | --- |
| \| YU MYC TARGETS DN \| \| --- \| \| NOJIMA SFRP2 TARGETS DN \| \| FOSTER INFLAMMATORY RESPONSE LPS UP \| \| SCHLOSSER MYC AND SERUM RESPONSE SYNERGY \| \| EBAUER TARGETS OF PAX3 FOXO1 FUSION UP \| \| DACOSTA UV RESPONSE VIA ERCC3 UP \| \| SCHLOSSER SERUM RESPONSE AUGMENTED BY MYC \| \| KEGG ANTIGEN PROCESSING AND PRESENTATION \| \| WAMUNYOKOLI OVARIAN CANCER LMP UP \| \| MULLIGHAN MLL SIGNATURE 1 UP \| \| BIOCARTA LYM PATHWAY \| \| APPEL IMATINIB RESPONSE \| \| REACTOME COSTIMULATION BY THE CD28 FAMILY \| \| SCHUHMACHER MYC TARGETS DN \| \| REACTOME MITOCHONDRIAL FATTY ACID BETA OXIDATION \| \| IVANOV MUTATED IN COLON CANCER \| \|  \| \| MILI PSEUDOPODIA HAPTOTAXIS UP \| \| KOBAYASHI EGFR SIGNALING 6HR UP \| \| SCHLOSSER SERUM RESPONSE DN \| \| TING SILENCED BY DICER \| \| CHEN HOXA5 TARGETS 9HR UP \| \| HAMAI APOPTOSIS VIA TRAIL UP \| \| SHEN SMARCA2 TARGETS UP \| \| WAMUNYOKOLI OVARIAN CANCER LMP DN \| \| GRABARCZYK BCL11B TARGETS UP \| \| KANG DOXORUBICIN RESISTANCE DN \| | \| 49 \| \| --- \| \| 23 \| \| 140 \| \| 23 \| \| 140 \| \| 230 \| \| 74 \| \| 41 \| \| 160 \| \| 254 \| \| 10 \| \| 27 \| \| 42 \| \| 6 \| \| 9 \| \| 5 \| \|  \| \| 350 \| \| 4 \| \| 488 \| \| 22 \| \| 134 \| \| 270 \| \| 256 \| \| 127 \| \| 44 \| \| 13 \| | \| 2.280 \| \| --- \| \| 2.526 \| \| 2.021 \| \| 2.821 \| \| 2.300 \| \| 3.000 \| \| 2.443 \| \| 3.625 \| \| 3.775 \| \| 2.619 \| \| 1.359 \| \| 2.824 \| \| 2.373 \| \| 1.780 \| \| 2.419 \| \| 1.330 \| \|  \| \| -7.807 \| \| -3.230 \| \| -4.312 \| \| -2.501 \| \| -4.018 \| \| -3.401 \| \| -6.440 \| \| -2.395 \| \| -2.689 \| \| -2.256 \| | \| 0.001 \| \| --- \| \| 0.010 \| \| 0.016 \| \| 0.009 \| \| 0.018 \| \| 0.009 \| \| 0.026 \| \| 0.002 \| \| 0.001 \| \| 0.015 \| \| 0.003 \| \| 0.030 \| \| 0.026 \| \| 0.006 \| \| 0.010 \| \| 0.024 \| \|  \| \| 2.3E-11 \| \| 9.3E-09 \| \| 0.000 \| \| 0.047 \| \| 0.000 \| \| 0.003 \| \| 2.5E-10 \| \| 0.031 \| \| 0.016 \| \| 0.002 \| | \| 0.037 \| \| --- \| \| 0.130 \| \| 0.174 \| \| 0.129 \| \| 0.183 \| \| 0.126 \| \| 0.219 \| \| 0.039 \| \| 0.031 \| \| 0.173 \| \| 0.056 \| \| 0.235 \| \| 0.218 \| \| 0.095 \| \| 0.136 \| \| 0.213 \| \|  \| \| 2.5E-08 \| \| 2.0E-06 \| \| 0.009 \| \| 0.292 \| \| 0.006 \| \| 0.063 \| \| 1.0E-07 \| \| 0.237 \| \| 0.174 \| \| 0.039 \| | \| 5.014 \| \| --- \| \| 4.882 \| \| 4.539 \| \| 3.974 \| \| 3.924 \| \| 3.366 \| \| 3.274 \| \| 3.041 \| \| 2.883 \| \| 2.630 \| \| 2.629 \| \| 2.360 \| \| 2.277 \| \| 1.633 \| \| 1.424 \| \| 1.215 \| \|  \| \| -5.562 \| \| -5.011 \| \| -4.924 \| \| -3.307 \| \| -3.229 \| \| -3.041 \| \| -2.664 \| \| -2.368 \| \| -2.093 \| \| -1.893 \| | \| 0.017 \| \| --- \| \| 0.002 \| \| 0.003 \| \| 0.020 \| \| 0.003 \| \| 0.020 \| \| 0.006 \| \| 0.040 \| \| 0.020 \| \| 0.036 \| \| 0.020 \| \| 0.035 \| \| 0.012 \| \| 0.031 \| \| 0.035 \| \| 0.000 \| \|  \| \| 6.1E-09 \| \| 0.031 \| \| 3.4E-05 \| \| 0.012 \| \| 0.002 \| \| 0.011 \| \| 0.003 \| \| 0.003 \| \| 0.042 \| \| 0.040 \| | \| 0.161 \| \| --- \| \| 0.042 \| \| 0.049 \| \| 0.179 \| \| 0.053 \| \| 0.178 \| \| 0.087 \| \| 0.258 \| \| 0.179 \| \| 0.248 \| \| 0.178 \| \| 0.246 \| \| 0.139 \| \| 0.230 \| \| 0.244 \| \| 0.005 \| \|  \| \| 7.7E-07 \| \| 0.231 \| \| 0.002 \| \| 0.139 \| \| 0.042 \| \| 0.129 \| \| 0.052 \| \| 0.056 \| \| 0.263 \| \| 0.259 \| |

All gene sets were statistically significant with Z-score > 1.5 in either direction, p<0.05 and false discovery rate <0.3. See Materials and Methods for additional details.
